# Effect of Pullulanase Debranching Time Combined with Autoclaving on the Structural, Physicochemical Properties, and In Vitro Digestibility of Purple Sweet Potato Starch

**DOI:** 10.3390/foods11233779

**Published:** 2022-11-23

**Authors:** David Mahoudjro Bodjrenou, Xin Li, Wei Chen, Yi Zhang, Baodong Zheng, Hongliang Zeng

**Affiliations:** 1 College of Food Science, Fujian Agriculture and Forestry University, Fuzhou 350002, China; 2 Fujian Provincial Key Laboratory of Quality Science and Processing Technology in Special Starch, Fujian Agriculture and Forestry University, Fuzhou 350002, China; 3 Key Laboratory of Subtropical Characteristic Fruits, Vegetables and Edible Fungi Processing (Co-Construction by Ministry and Province), Ministry of Agriculture and Rural Affairs, Fuzhou 350002, China

**Keywords:** purple sweet potato resistant starch, pullulanase debranching, autoclave treatment, structural characteristics, physicochemical properties

## Abstract

The effects of pullulanase debranching combined with autoclaving (PDA) at various debranching times (0 h, 5 h, 10 h, 15 h, 20 h, and 25 h) and 121 °C/20 min of autoclave treatment on the structural and physicochemical characteristics of purple sweet potato (Jinshu No.17) starch were investigated. The results indicated that the native starch (NS) was polygonal, round, and bell-shaped with smooth surfaces. After debranching treatment, the surface of the starch samples became rough and irregular. The molecular weight became smaller after treatments. X-ray diffraction C-type pattern was transformed into a B-type structure in treated samples with increased relative crystallinity. ^13^C NMR indicated an increased propensity for double helix formation and new shift at C1, 3, 5 region compared to NS. The apparent amylose content was 21.53% in the NS. As the swelling power decreased, the percentage of soluble solids increased and different thermal properties were observed. A higher yield of the resistant starch (RS) was observed in all treated starch except PDA 25 h. The findings of our study reveal that a combination of pullulanase debranching time (15 h) and autoclaving (121 °C for 20 min) is a great technique that can be used to produce a higher amount of resistant starch in the Jinshu No.17 starch.

## 1. Introduction

Purple sweet potato, *Ipomoea batatas* (L.) Lam., is a dicotyledonous sweet potato cultivar that belongs to the Convolvulaceae family [1,2]. Purple sweet potato is gaining popularity due to its nutritional composition, bright color, and culinary versatility. Although purple sweet potato is a promising source of anthocyanins for the food industry, a large amount of starch is wasted as a byproduct [3,4]. It has been considered an excellent source of carbohydrates (≥85%) and phytochemical components (total antioxidant activity: 27.20 µmol TG/g, total phenol content: 0.95 mg CAE/g fw, anthocyanin content: 0.53 mg/g fw, and β-Carotene: 46.90 µg/g fw) which are important for human survival [5]. Purple sweet potatoes typically have more than 40% total dry weight starch, whereas fresh tubers have between 12 and 30% starch. While the isolation of starch from purple sweet potato has been widely investigated [6,7,8], the production of its resistant starch (RS) has received little attention. Current studies demonstrate that the structural, physicochemical, and functional characteristics of starch are closely related to the breeding line, variety, and growth conditions of purple sweet potato [6,8,9].

Starch, a natural micro-crystalline biopolymer, is the most abundant reserve carbohydrate from plant organs such as seeds, fruits, leaves, tubers, and roots [10]. Starch is composed of linear amylose and branching amylopectin molecules. The linear chain of amylose comprises α-(1–4)-linked D-glucose units, while amylopectin is made up of smaller chains of α-(1–4)-linked D-glucan with α-(1–6)-linked D-glucose branches [11]. According to their bioavailability, rate, and extent of digestion or glucose release, starch is classified as rapidly digestible starch (RDS), slowly digestible starch (SDS), and RS [12]. The RDS refers to the glucose level produced within 20 min after digestion and is swiftly and extensively digested in the small intestine. SDS is slowly and completely digested in the small intestine, with a glucose release time of 20 to 120 min [13]. In the large intestine, RS resists digestion and serves as a substrate for bacterial fermentation, which creates short-chain fatty acids, lactic acid, and gas, all of which are beneficial to colon health [14]. After 120 min of incubation, the total RS minus glucose percentage is released. RS can be categorized into five types: RS1, RS2, RS3, RS4, and RS5. RS1 (physically inaccessible) and RS2 (native B- and C-type polymorphic starch granules or ungelatinized starch) lose their resistance during food processing. When heated over 100 °C, RS3 (retrograded or crystalline starch), which is formed through the processes of gelatinization and subsequent recrystallization, is stable. RS4 (starch chemically modified) can be prepared by etherification, esterification, and cross-linking, which is indigestible by digestive enzymes. RS5 (amylose-lipid complexes) can be formed by assuming that the aliphatic part of the lipid is included inside the amylose helix in the case of amylose-lipid complexes, which requires higher temperatures for gelatinization, and the polar group of lipid lies outside, which is too large to be included [13]. Of particular interest to most scientists is RS3 which is retrograded amylose or crystalline non-granule starch formed through the reorganization of starch chains, because of their thermal stability. Physical processes, including autoclave, heat, and moisture, can promote the formation of RS3 [14,15]. The amylose content can affect the formation of RS3.

Currently, diverse modification approaches, such as physical, chemical, and enzymatic treatments have been reported to improve the RS content in starch. Among them, enzyme debranching of starch is the most common approach used to increase the RS3 content. Debranching starch, also called short-chain amylose or short linear glucan, is performed by treating starch paste with pullulanase or isoamylase, which causes the formation and reorganization of linear short-chain glucans under different storage conditions [16]. Pullulanase or isoamylase debranching causes reorganization of short linear glucans, generates the linear short chains released from amylopectin, alters the physicochemical properties and digestibility of starch, and assists with the formation of RS. Purple sweet potato (Xinyin No.1) starch has been pullulanase debranched combined with heat-moisture treatment and recrystallized to obtain RS (17.16%) which was higher compared to the heat moisture treatment (14.23%), and native starches (5.02%) [17]. Huang et al., [18] reported that the debranching heat-moisture treatment of sweet potato starches, RS, and SDS increased as compared to debranching treatment. From sweet potato starch, a higher RS (28.76%) content was observed in debranched starches stored at 4 °C [19]. In addition, most researchers have investigated the influence of debranching and heat-moisture treatment on the structural characteristics of starches from wheat [20], cassava [21], and waxy maize [22,23,24]. The above studies mainly investigated the effect of the enzyme debranching conditions (time and concentration) on RS formation. The starch from purple yam was autoclaved and then debranched with a pullulanase, according to a study [25]. This significantly increased the resistant starch yield. However, there are few reports and detailed descriptions available on the formation of RS, the changes in crystallinity, and the ordered structure within purple sweet potato starch in the hydrolysis procedure of PDA, to explain the characteristic phenomenon during purple sweet potato digestibility.

Therefore, the objective of the present study was to investigate the effect of pullulanase debranching time combined with autoclaving on the structural and physicochemical properties, and in vitro digestibility of purple sweet potato starch. The structural characteristics such as morphology observed by scanning electron microscopy (SEM), X-ray diffraction (XRD), and Fourier transform infrared spectroscopy (FTIR) of starches were analyzed. The physicochemical characteristics including amylose content, swelling power and solubility, thermal property, and in vitro digestibility of starches were determined. The findings may assist in the production of new food ingredients used as RS with improved health benefits.

## 2. Materials and Methods

### 2.1. Plant Material and Chemical Reagents

The purple sweet potato (Jinshu No.17) variety was grown in an experimental field in Fuzhou city (Fujian, China) and harvested in October 2020. α-amylase (50,000 U/g) and glucoamylase (100,000 U/g) were acquired from Yuanye Bio-Technology Co., Ltd. (Shanghai, China), while pullulanase (≥1000 npun/g) was obtained from Shanghai Aladdin Biotechnology (Shanghai, China). All other chemicals and reagents used in this experiment were analytical grades.

### 2.2. Extraction of Starch from Jinshu No.17

The starch was extracted as described by Gou et al., [26] with slight modifications. Briefly, fresh Jinshu No.17 tubers were carefully washed with water to remove all the dirt and soil, peeled and cut into small pieces. The small pieces were immediately homogenized with distilled water and grounded in a blender for approximately 1~1.5 min after adding distilled water (Jinshu No.17 and distilled water ratio was 1:3). The mixture was filtered through a 100-mesh filter cloth and the filtrate was washed after 6 h of sedimentation to discard the precipitated part of the surface fat. The suspension was discarded, and the sediment obtained was rinsed with distilled water three times until the supernatant became clear. The final sediment was dried at 45 °C for 36 h in a drying oven. Afterwards, the resulting product was ground and passed through 100-mesh sieves and stored for further analysis.

### 2.3. Debranching Treated and Debranching Combined Autoclave Treated Starch

Preparation of pullulanase debranching starch was performed by a combination of the methods described by Reddy et al. [27] and Surendra Babu & Parimalavalli [19] with some modifications. Jinshu No.17 starch (50 g, dry basis) was dispersed in distilled water (30% *w*/*v*) and the pH value was adjusted to 4.5–5.0 with Na_2_HPO_4_ (0.2 mol/L) buffer solution. The mixture was gelatinized in a water bath under stirring at 80 °C for 15 min. The gelatinized starch was cooled to 60 °C, and debranched by pullulanase (≥1000 npun/g) at the concentration of 10 npun/g of starch. After 0 h, 5 h, 10 h, 15 h, 20 h, and 25 h of reaction, the mixture level was adjusted to 250 mL with distilled water and the dispersion was heated in boiling water at 95 °C for 15 min to stop the pullulanase reaction. The debranched starch dispersion was then cooled to room temperature before being autoclaved for 20 min at 121 °C, re-cooled to room temperature, and stored at 4 °C for 24 h for retrogradation. The resultant was dried at 60 °C for 16 h, ground, and pass through a 100-mesh sieve. The obtained pullulanase debranching time (0 h, 5 h, 10 h, 15 h, 20 h, and 25 h) combined with autoclaving treatments starch was labeled PDA 0 h, PDA 5 h, PDA 10 h, PDA 15 h, PDA 20 h, and PDA 25 h, respectively.

### 2.4. Morphology Observation and Granule Size Analysis

A field scanning electron Microscopy system (*/Nova NanoSEM 230, FEI Czech Republic S.R.O. Co, Ltd., Hillsboro, OR, USA) was applied to inspect the morphology of the native and modified purple sweet potato starch. The dried starch samples were sputtered with a layer of gold prior to imaging. A micrograph of each sample was taken at an accelerating voltage of 8.00 Kv with 5000× magnification [28]. The granule sizes of starch (0.5 g) were analyzed using a laser diffraction particle size analyzer Malvern Master sizer Hydro 3000 (UK) following the method of Zhang et al. [8].

### 2.5. Gel Permeation Chromatography (GPC)

The molecular weight distributions of the native and PDA were measured using gel permeation chromatography (GPC) according to the method described by Ji et al. [29] with some modifications. Briefly, the starch samples (1.0 mg/mL) were completely dissolved in dimethyl sulfoxide (DMSO: 100 μL) and constantly stirred in a boiling water bath for 24 h. After passing through a 2 μm organic filter, the starches were injected by an autosampler into a PL-GPC 50 system (Agilent PL-GPC 50, UK) with PL gel Olexis column (7.5 × 300 mm) and differential refractive index detector. The column oven temperature was maintained at 80 °C, but at 40 °C overnight. The dextran standards with molecular weights ranging from 6 × 10^7^ g/mol to 17 × 10^7^ g/mol were used for molecular calibration.

### 2.6. X-ray Diffraction Pattern

The crystalline structure of starch samples was analyzed using an X-ray powder diffractometer (XRD) (Ultima IV, Tokyo, Japan) according to the method of Yong et al. [1] with some modifications. The diffraction was operated at 40 kV and 40 mA with Cu Kαbeta filter radiation. The scanning region was 5–40° (2θ) with a scanning speed of 1 °/min. The relative crystallinity percentage is calculated using the diffraction ratio of the peak area to the total area.

### 2.7. Solid-State 13C CP/MAS NMR Spectroscopy

Sample powder (0.3 g) was analyzed using a ^13^C nuclear magnetic resonance spectrometer (AVANCE Ш 500, Bruker Ltd., Biospin GmbH, Fällanden, Switzerland), using a MAS VTN 4 mm probe. The resonance frequency was 125.7 MHz, the spectral width 4 kHz, the acquisition time 50 ms and the time domain 2k. Each spectrum was scanned 2400 times. The signal intensity of the *C*1 region reflected the crystallized region of starch, the intensity of the *C*4 region reflected the amorphous region of starch, and the intensity of the *C*2, 3, 5 region reflected the degree of freedom of the amylose chains, as determined previously by Zheng et al. [30].

### 2.8. Determination of Apparent Amylose Content

A colorimetric iodine affinity procedure (Thermo Fisher Multiskan GO, Waltham, MA, USA) multi-function microplate reader method described by Wang et al. [31] with some modifications was used to determine the amylose content. Briefly, 0.01 g of starch sample was accurately weighed and 100 μL of ethanol and 900 μL of sodium hydroxide (NaOH) solution were added, mixed well, boiled in a water bath for 10 min, allowed to cool, and diluted to 10 mL. Afterwards, 0.5 mL of the mixture was introduced into the centrifuge tube (15 mL), 0.1 mL of acetic acid (1N) and 0.2 mL iodine solution, diluted to 10 mL, stood at room temperature for 10 min, and the absorbance was measured using a spectrophotometer at 720 nm. The amylose standard was obtained using Rice-Determination of amylose content (GB/T 15683-2008) (https://connecting-asia.org/wp-content/uploads/2017/01/Rice-%E2%80%93-Determination-of-amylose-content.pdf (accessed on 30 September 2022)). The apparent amylose content was calculated as follows:

(1) $$\mathrm{Apparent}\;\mathrm{amylose}\;\mathrm{content}\;\left(\%\right)=\frac{C}{M}\times 100$$

where C is the result calculated according to the standard curve; M is the actual weighed mass (usually 10 mg); 10 is the correction factor.

### 2.9. Differential Scanning Calorimetry (DSC)

A 3 mg starch sample was mixed with 9 µL distilled water and sealed in an aluminum pan. After equilibrating for 2 h at room temperature, samples were heated from 30 to 190 °C at a rate of 10 °C/min using a differential scanning calorimetry (DSC, 200-F3, Netzsch, Germany) [8].

### 2.10. Fourier Transform Infrared (FT-IR) Spectroscopy

A Nicolet Avatar 360 FR-IR spectrometer (Thermo Fisher Scientific, Waltham, MA, USA) was used to record the spectrum of native and modified starch samples at room temperature. Before measurement, the starch sample (0.2 g) was ground with KBr powder (150 mg) for 10 min under an infrared lamp and pressed into tablets. The average of 32 scans at a resolution of 4 cm^−1^ was used to calculate a scanning region ranging from 500 to 4000 cm^−1^ [8].

### 2.11. Swelling Power and Percentage of Soluble Solids

Starch sample (0.5 g) was mixed with 25 mL of distilled water and heated at 65, 75, 85, and 95 °C in a water bath for 20 min and stirred every 5 min. The samples were cooled to room temperature, and the suspension was centrifuged at 3500 rpm for 20 min. The supernatant and sediment samples were separated and weighed, and the swollen starch sediment was weighed after an aliquot of the supernatant was evaporated at 105 °C for 2 h [32]. The equation used to calculate the swelling power (SP) and the percentage of soluble solids (SS) were as follows:

(2) $$\mathrm{SP}\;\left(\frac{g}{g}\right)=\mathrm{Sw}/\left(\mathrm{Ms}-\mathrm{SS}\right)$$

where Sw is the sediment weight, g; Ms is the mass of starch in origin dried, g;

(3) $$\mathrm{SS}\;\left(\frac{g}{g}\right)=\mathrm{Mds}/\mathrm{Ms}\times 100$$

where Mds is the mass of dry solids in sediment, g; Ms is the mass of the starch in the origin dried, g.

### 2.12. Determination of In Vitro Digestibility

The Jinchu No.17 starch fractions in the samples were determined by previously described method of Thanh et al. [9] with some modifications. In brief, 2 g starch samples were weighed and dissolved in acetate buffer (10 mL, pH 5.2), heated in a boiling water bath for 20 min, and allowed to stand and cool at room temperature. Afterwards, 10 mL of mixed enzyme solution (2.5 g of pancreas α-amylase (100 U/mL) and 0.4 mL amyloglucosidase (100,000 U/mL)) mixed in 100 mL phosphate buffer saline (pH 7.2) was added and incubated at 37 °C (160 rmp/min) for 0, 20, 40, 60, 90, 120, and 150 min. Aliquots (0.1 mL) were taken from each tube at each designated incubation time and mixed with 0.9 mL 95% ethanol solution for 1 min. The released glucose content was determined by the GOPOD method (0.1 mL sample + 3 mL GOPOD solution, water bath at 45 °C for 20 min) and determine the absorbance value at 510 nm. The fractions of RDS, SDS, and RS were calculated as follows:

(4) $$\mathrm{RSD}\;\left(\%\right)=\left[\left(\mathrm{Gl}20-\;\mathrm{Gl}0\;\right)\times \frac{0.9}{\mathrm{SW}}\right]\times 100$$

(5) $$\mathrm{SDS}\;\left(\%\right)=\left[\left(\mathrm{Gl}120-\;\mathrm{Gl}20\;\right)\times \frac{0.9}{\mathrm{SW}}\right]\times 100$$

(6) $$\mathrm{RS}\;\left(\%\right)=\left[100-\left(\mathrm{RSD}+\mathrm{SDS}\right)\right]\times 100$$

where Gl0, Gl20, and Gl120 are the weights of glucose produced at 0, 20, and 120 min, respectively. SW is the weight of the starch sample; RDS, SDS, and RS are the percentages of rapidly digestible starch, slowly digestible starch, and resistant starch, respectively.

### 2.13. Statistical Analysis

Graphs were constructed in OriginPro 2021 (OriginLab Corporation, Northampton, MA, USA). IBM SPSS (version 26, Chicago, IL, USA) was used for the experimental data analysis, and one-way ANOVA was performed to test for significant differences (*p* < 0.05, significant difference). All experiments were repeated at least in triplicate measurements.

## 3. Results and Discussion

### 3.1. Morphology and Size Distribution

**Figure 1:**
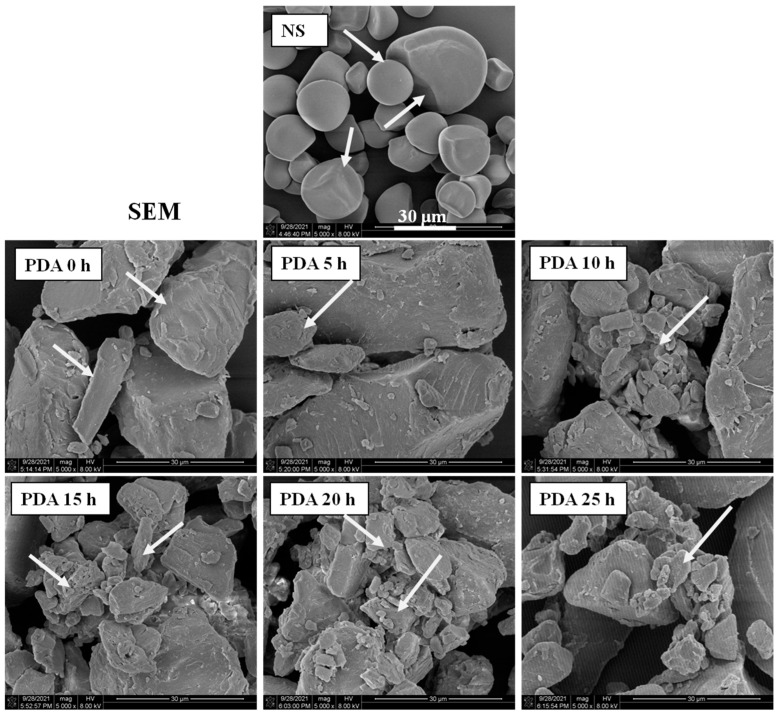
Scanning electron microscopy (SEM) images of native (NS) and debranched in different treatment times combined with autoclaved starches (PDA 0 h, PDA 5 h, PDA 10 h, PDA 15 h, PDA 20 h, and PDA 25 h). The magnification of images was 5000.

**Figure 2:**
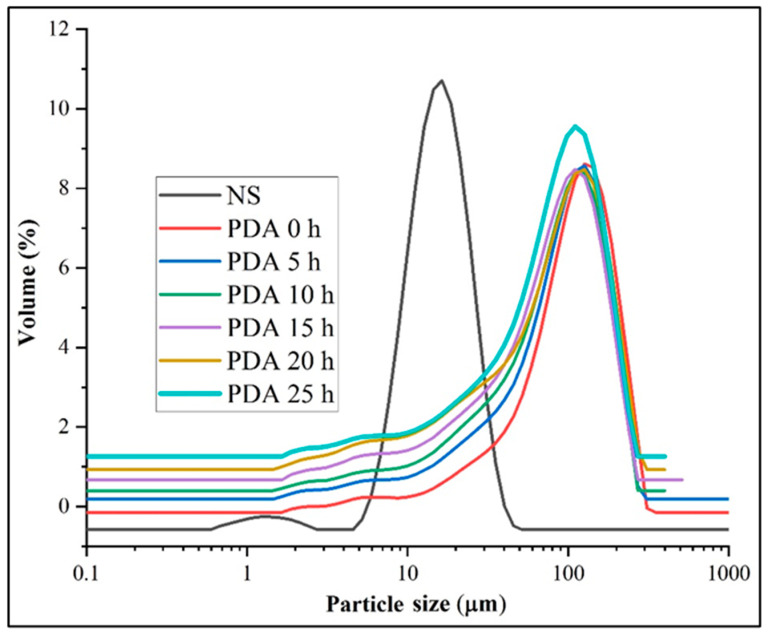
Particle size of purple sweet potato native and debranched at different times combined with autoclaved starches. NS, PDA 0 h, PDA 5 h, PDA 10 h, PDA 15 h, PDA 20 h, and PDA 25 h indicate native starch, pullulanase debranching in different treatment times combined with autoclaving starch.

The morphology of native and debranching combined with autoclaving starches prepared from Jinshu No.17 is shown in Figure 1. Native starch (NS) granules were oval, round, polygonal shapes without fissures on the surface, and smooth structure, which is in agreement with previous reports on purple sweet potato starch granules [17]. After treatment, the granule structure vanished. Starch samples had a rough surface, collapsed, and irregularly shaped structures were observed after modification. The native granule structure of purple sweet potato was destroyed and became much larger and more tightly packed after being debranched, autoclaved, and retrograded. This was consistent with previous findings in chickpea starch [33], Xinyin No.1 starch [17], lotus seed starch [34], and red kidney bean starch [35]. This proves that pullulanase has an enzymatic hydrolysis effect on purple sweet potato starch, which alters the original structure of starch and has a relationship with debranching time. As shown in Figure 2 and Table 1, the particle size of starches and their distribution profile changed significantly after PDA treatment. The NS showed bimodal size distributions with small particle sizes of 0.59–2.75 μm and large particle sizes of 4.58–51.80 μm. Moreover, the diameter of Jinshu No.17 starch granules was comparable to those from previous studies [36,37]. The treated starches showed large particle sizes of 1.65–310 μm. Compared to PDA 0 h, D [3,2], D [4,3], Dx (10), Dx (50), and Dx (90) decreased when the debranching time increased, but an increase was observed at PDA 20 h and PDA 25 h. This result indicated that the PDA treatment considerably increased the diameters of particle size.

### 3.2. Molecular Weight Distribution

Gel permeation chromatography (GPC) technique was used for analyzing the polymer of starch samples to determine their molecular weight (Mw), number average (Mn), and polydispersity values [38]. The molecular weight distributions (Mw) of Jinshu No.17 starch at the different debranching times combined with autoclaving treatment are shown in Table 2. A high Mw (8.54 × 10^6^ g/mol) was observed in the NS. After PDA treatment, the samples showed a moderate and broad molecular distribution with lower Mn and Mw after modification under different treatment times compared to NS. This indicates that purple sweet potato starch has been broken down into low-polymerization debranching starch. The polydispersity values (PD) of all treated samples increased, indicating a larger molecular weight dispersion following debranching times. This indicates that the purple sweet potato starch was digested by pullulanase to prepare starch with a smaller molecular weight. The degraded products of the starch samples entered the gel and were eluded together to form an amylose peak, thereby increasing the percentage of resistant starch [39]. After 25 h of debranching, the starch sample recovered due to degradation, and the raised Mw value was attributed to structural damage or disruption of the starch, which reduced the impressibility of the starch to enzyme digestion. This damage may result in a rise in RDS and a fall in RS.

### 3.3. Crystalline Characteristics

**Figure 3:**
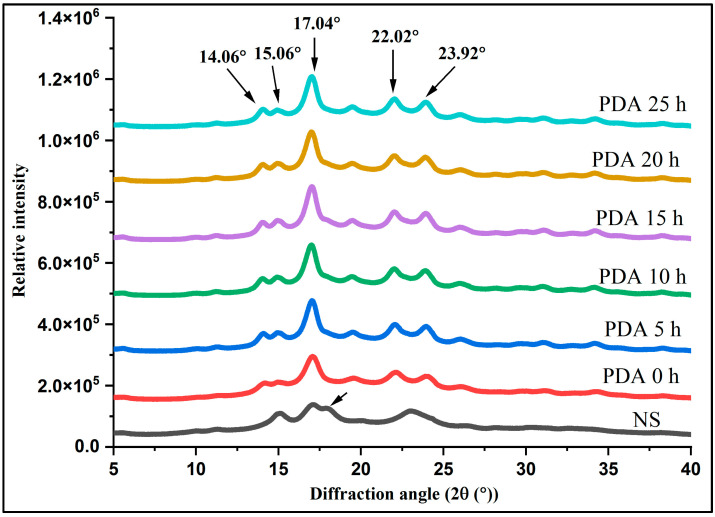
X-ray diffraction patterns of native and debranched combined with autoclave treatment starches. NS, PDA 0 h, PDA 5 h, PDA 10 h, PDA 15 h, PDA 20 h, and PDA 25 h indicate native starch and pullulanase debranching in different treatment times combined with autoclaving starch.

Figure 3 shows the XRD spectra of native and PDA starch samples of Jinshu No.17. The NS exhibited strong diffraction peaks (2θ) at 15.12° and 22.96°, as well as unresolved doublet peaks at 17.02 and 17.88° (2θ), and a small peak at around 5.49° (2θ), indicating that the crystalline pattern of this starch was characterized as typical C-type (Table 3). The observation agrees with previous studies that reported that seven purple sweet potatoes’ starches were C_a_-type crystalline polymorph [1,8]. However, Trung et al. [9] reported that NS from white, yellow and purple sweet potatoes exhibited an A-type polymorph pattern. Kim et al. [6] reported the crystalline types of starches isolated from Korean purple sweet potatoes, Shinjami and Borami, were respectively C_b_-type and A-type. After samples were subjected to pullulanase debranching time combined with autoclaved treatment and retrograded, all the X-ray diffraction patterns of starches was transformed to a B-type structure with a principal sharp peak (2θ) at 17° and some small additional peaks at around (2θ) values of 14.96, 22.12, and 23.92° which were characterized as B-type crystallinity [40]. The change in the crystal structure indicates that the original crystal structure of starch granules was damaged. This result was in accordance with previous studies, which showed that the debranched starch and autoclaved starch tended to exhibit the typical B-type crystalline polymorph [41,42]. As shown in Table 3, the relative crystallinity of NS was 26.46%, which was comparable to those of purple sweet potato varieties in the previous report [8]. The treated starches showed increasing crystallinity values with the debranching time compared to their counter NS. This is because hydrolysis increases the number of linear chains, which are less branched and have a lower molecular mass, resulting in a more crystalline structure. The finding indicates that the reassociation of linear chains released by pullulanase at different treatment times can form more ordered structures after autoclaving. According to the result, pullulanase debranching combined with the autoclave causes a higher relative crystallinity as debranching times increase, which could be explained by a higher RS content after debranching [19].

### 3.4. Ordered Structural Characteristics Determined by 13C CP/MAS NMR

**Figure 4:**
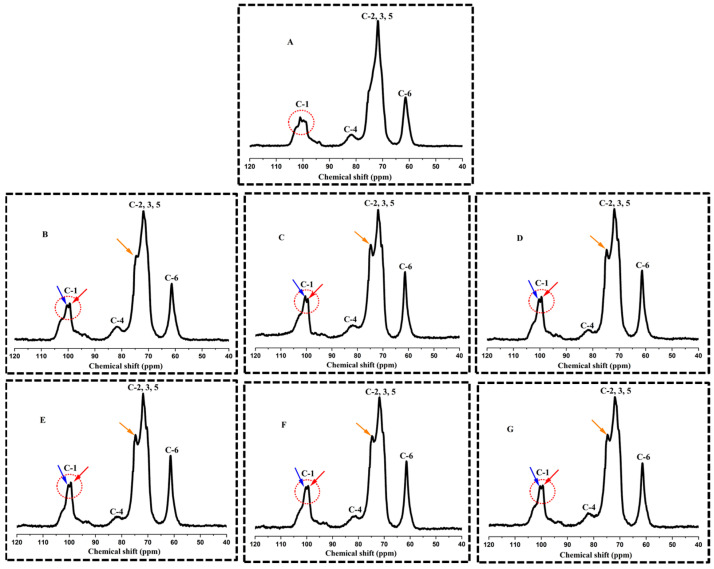
Solid state ^13^C nuclear magnetic resonance spectroscopy characteristics of native starch (NS) and debranched starches (PDA) at different treatment times combined with autoclaved. (**A**) indicated NS; (**B**–**G**) indicate PDA 0 h, PDA 5 h, PDA 10 h, PDA 15 h, PDA 20 h, and PDA 25 h, respectively.

The nuclear magnetic resonance spectra of pullulanase debranching with autoclave treatment on purple sweet potato starch were assigned as described previously and are displayed in Figure 4. According to the literature, the shift at 94–105 ppm was associated with C-1, the shift at 80–84 ppm was attributed to amorphous domain of C-4, the overlapping shifts at 68–78 ppm were associated with C-2, C-3, and C-5, the shift at 58–65 ppm was related to C-6 [41]. In the C1 region, the spectra of purple sweet potato starch showed inconspicuous triplet peaks (at 102.93, 100.97, and 99.10 ppm), that exhibit characteristics of both the A-type and B-type crystal structure. These observations were also in connection with those of XRD analysis and a study published by Huimin et al. [1]. The C-1 resonance exhibits B-type conformation with two peaks (100.53 and 99.34 ppm) after pullulanase debranching treatment, corresponding to the non-identical sugar residues of amylose and amylopectin. While the C-4 region provides details on the amorphous state, the C-2, C-3, and C-5 regions primarily showed the B-type double helices with new peaks around 74.87 ppm caused by the residues of free amylose. The obtained results suggest that the combined treatments of pullulanase debranching and autoclaving on purple sweet potato starch which induced the most intense molecular chain degradation, had a greater influence on promoting the formation of double helix structure.

### 3.5. Apparent Amylose Content

The apparent amylose content (AC) of native purple sweet potato starch was found to be 21.53% (Table 3). The amylose in debranched followed by autoclaved samples was significantly decreased by 4.98% (PDA 0 h), 10.16% (PDA 5 h), 12.20% (PDA 10 h), 13.19% (PDA 15 h), 12.63% (PDA 20 h), and 12.54% (PDA 25 h) compared to the native starch. The results revealed that the pullulanase debranching times and treatment conditions may have influenced the AC by lowing its content with increasing times. A previous study reported that more than half of a percent of the debranching in starch occurred during the first four hours of the debranching treatment [31]. As the duration of debranching increased, the debranching became exceedingly slow until it attained a constant value. On the other hand, starch-iodine complexes require a certain chain length to form, and it is difficult for smaller chains to form stable complexes with iodine [33]. Long-term debranching contributes to a loss in the process when determining AC using the iodine-amylose complex formation method. This may be the reason for the interpretation to understand that AC does not show a continuous increase during the whole process.

### 3.6. Thermal Parameters Analysis

The DSC of the NS and PDA of Jinshu No.17 starch samples is shown in Table 4. The NS of Jinshu No.17 exhibited a clear gelatinization peak at 75.80 °C (Tp), 59.00 °C for onset temperature (To), and 84.73 °C for conclusion temperature (Tc), with an endothermic enthalpy (ΔH) of 11.47 J/g. The thermodynamic characteristic values of To, Tp, Tc, and ΔH of the treated starch samples ranged from 48.30–78.56 °C, 72.30–95.50 °C, 95.33–127.20 °C, and 9.68–13.23 J/g, respectively, and increased after 5 h, 10 h, 15 h, and 20 h when compared to the 0 h starch sample. The starch transition temperatures (To, Tp, Tc) and ΔH had significantly decreased after 25 h. This could occur because the enzyme repair increases the orderly orientation and arrangement of the starch chain, reducing the structural flexibility of the starch, and affecting the modified starch to form better crystal stability. However, the gel temperature range of enzymatic disintegration and autoclave treatment of starch narrowing is due to the difference increase in To-Tc. As the enzyme reaction time increases, ΔH is increase slightly, with higher values after 15 h (PDA 15 h). In the present study, it was discovered that when compared to PDA 0 h, starch sample by PDA 15 h with the higher RS content (70.14%) had higher ΔH values (13.23 J/g). This finding is consistent with other studies on sweet potato starch after debranching [18]. The transition temperatures reflect the melting of amylopectin crystals with potential contributions from all crystal settling and are also associated with the formation of the RS crystalline structure during heating and cooling [19]. According to the findings, the starch samples solidifying at the early stages of debranching had a weak crystalline structure and a wide range of crystal size and perfection. The result suggested that debranching for a short period of time may be required to decompose the double helix structure of starch.

### 3.7. Fourier Transform Infrared (FT-IR) Analysis

**Figure 5:**
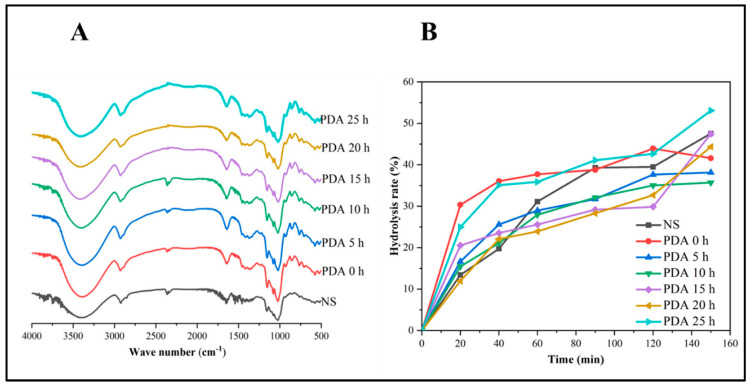
FT−IR spectra (**A**) and in vitro hydrolysis curves (**B**) of native and debranched starch samples. NS, PDA 0 h, PDA 5 h, PDA 10 h, PDA 15 h, PDA 20 h, and PDA 25 h indicate native starch and pullulanase debranching in different treatment times combined with autoclaving starch.

The FT-IR spectrum can reflect the short-range ordered structure in the starch external region through vibrational deviations upon interactions between native and modified starches. Figure 5A shows the FT-IR spectra of NS and PDA treatment of Jinshu No.17. FT-IR spectra of the NS show a typical absorption peak similar to a previous report [17]. As seen from FT-IR spectra, the NS and PDA show typical peaks at 3398 cm^−1^ for stretching vibration of O-H groups, at 2922 cm^−1^ for stretching vibrations of C-H and CH2, at 1645 cm^−1^ for H-O-H bending of water molecules adhered to NS and PDA at different times, at 1156 cm^−1^ for asymmetric stretching vibration of C-O-C, at 1026 cm^−1^ for symmetric stretching vibration of C-O-C. The characteristic peaks of both NS and PDA at approximately 3035–3710 cm^−1^ shifted to a higher frequency, indicating that pullulanase debranching treatment times with greater R spectra values promote higher levels of order in the external regions of the starch granule [27,43].

### 3.8. Swelling Power and Percentage of Soluble Solids

The swelling power and percentage of soluble solids of NS and PDA starches are presented in Table 5. On the whole, the swelling power of the starch samples showed a positive correlation with the temperature (65–95 °C). The swelling power of NS ranged from 28.52 to 30.32 g/g, which was comparable to the previous report [8]. After different debranching times combined with autoclaving treatment, a considerable reduction value of swelling power was registered. A similar tendency was previously reported on debranching sweet potato starch [19] and *Dioscorea alata* L [25]. It has been suggested that the higher ratio of amylose content and the presence of a stronger or more important number of intermolecular bonds can decrease starch swelling. As shown in Table 5, the water solubility of PDA treatment starches was a higher value than the NS (0.33–0.41 g/g with increasing temperature). The percentage of soluble solids are frequently used as an indicator of molecular component degradation. The increase in percentage of soluble solids may happen as the result of changes in molecular structure, or it may be an independent mechanism that causes starch components to flow out, causing carbohydrate leaching from related molecules [19]. Therefore, the combination of pullulanase debranching time with autoclaving improves starch percentage of soluble solids by decreasing the molecular mass of the starch sample.

### 3.9. In Vitro Digestion Property

The RDS, SDS, and RS content in the Jinshu No.17 NS and PDA starch samples are presented in Table 6 and Figure 5B. As shown in Table 6, the RDS, SDS, and RS of the NS were 13.42, 26.06, and 60.51%, respectively. The RS in the NS sample was significantly lower than the seven purple sweet potatoes genotypes (84.5–86.4%) [8] and significantly higher than the other seven genotypes (29.25–43.50%) [7]. The result was consistent with the previous report that purple sweet potato starch contained a large amount of RS which can be used for health benefits [25]. Compared with NS, except PDA 20 h, the treated starches (PDA 0 h, PDA 5 h, PDA 10 h, PDA 15 h, and PDA 25 h) exhibited higher RDS; while the SDS of all samples was lower than that of NS. After debranching, autoclaving, and storing at 4 °C of temperature for 24 h, the yield of RS was significantly increased compared to the NS and DPA0, when the pullulanase debranching times increased from 5 h to 20, could reach 70.14%. This result agreed with the previous reports on the formation of RS by debranching starch using pullulanase [17,19]. After 20 h of the debranching, the yield of RS began to decrease, indicating that amylopectin generated less short linear molecules to realign a new crystalline structure that can undergo retrograde processing after 20 h of debranching. The drop in RS and increase in RDS at PDA 0 h and PDA 25 h was attributed to starch structural degradation or disruption, as starch is rapidly digested in the small intestine.

## 4. Conclusions

The combination of pullulanase debranching time and autoclaving (PDA) has a significant effect on the structural, physicochemical characteristics, and in vitro digestibility of purple sweet potato starch. Compared with NS, the morphology of starch structure was destroyed with significantly different particle sizes after all debranching treatment times. Different decreases in the molecular weight were observed and the XRD revealed a change from C-type to B-type crystalline pattern when debranched and autoclaved, thus improving the regularity of the crystallites by enhancing RS yield. Analysis using NMR indicated an increase of the double helix and higher degree of freedom of amylose chains. The apparent amylose content and the thermal property of starch were greatly influenced by the duration of PDA. The FT-IR showed that there was no more change between the NS and PDA samples. The swelling power increased with the temperature and decreased with the debranching times, while the percentage of soluble solids increased. Higher RS content (70.14%) was obtained after 15 h of pullulanase debranching combined with autoclaving (121 °C/20 min). In comparison to the starch sample at 15 h, the damage after 20 h decreased RDS, increased SDS, while RS was low. The results demonstrated an increase in the RS, but it was not stable across treatment times. Pullulanase debranching times combined with autoclaving is a promising green and clean technique for the preparation of RS3. Therefore, purple sweet potato starch produced with PDA could be used as an ingredient in foods and industrial products to provide biological or physical functionalities.

## Figures and Tables

**Table 1:** Particle size of native and debranched combined with autoclaved starches.

Samples	Particle Size (μm)				
	D [3,2]	D [4,3]	Dx (10)	Dx (50)	Dx (90)
NS	12.04 ± 0.05 ^g^	17.51 ± 0.01 ^f^	9.04 ± 0.05 ^f^	16.44 ± 0.05 ^f^	28.14 ± 0.05 ^e^
PDA 0 h	51.40 ± 0.00 ^a^	119.07 ± 0.11 ^e^	30.01 ± 0.00 ^a^	114.01 ± 0.01 ^a^	214.07 ± 0.11 ^a^
PDA 5 h	40.50 ± 0.00 ^b^	105.03 ± 0.05 ^a^	22.04 ± 0.05 ^b^	99.87 ± 0.11 ^b^	193.33 ± 0.57 ^bc^
PDA 10 h	38.44 ± 0.05 ^d^	98.73 ± 0.22 ^b^	20.32 ± 0.03 ^c^	93.11 ± 0.00 ^c^	185.33 ± 0.57 ^c^
PDA 15 h	35.64 ± 0.05 ^e^	93.94 ± 0.05 ^d^	17.54 ± 0.05 ^d^	88.17 ± 0.28 ^e^	179.34 ± 0.57 ^d^
PDA 20 h	32.93 ± 0.05 ^f^	98.37 ± 0.55 ^b^	15.10 ± 0.00 ^e^	91.73 ± 0.23 ^d^	191.37 ± 0.55 ^b^
PDA 25 h	40.00 ± 0.01 ^c^	97.34 ± 0.57 ^c^	21.73 ± 0.57 ^b^	91.87 ± 0.11 ^d^	179.34 ± 0.57 ^d^

Data are mean ± standard deviations, *n* = 3. Values in the same column with different letters are significantly different (*p* < 0.05); The D [3,2] and D [4,3] are the surface-weighted and volume-weighted mean diameters, respectively; Dx (10), Dx (50), Dx (90) are the measured size value of 10%, 50%, and 90% of the particle size; NS, PDA 0 h, PDA 5 h, PDA 10 h, PDA 15 h, PDA 20 h, and PDA 25 h indicate native starch and pullulanase debranching in different treatment times (h) combined with autoclaving starch.

**Table 2:** Molecular weight of native and debranched combined with autoclaved starches.

Samples	Mn (g/mol)	Mw (g/mol)	PD
NS	5.74 × 10^6^	8.54 × 10^6^	1.49
PDA 0 h	1.98 × 10^6^	4.37 × 10^6^	2.20
PDA 5 h	1.68 × 10^6^	3.46 × 10^6^	2.06
PDA 10 h	1.32 × 10^6^	2.89 × 10^6^	2.18
PDA 15 h	1.49 × 10^6^	3.81 × 10^6^	2.55
PDA 20 h	1.39 × 10^6^	3.60 × 10^6^	2.58
PDA 25 h	2.15 × 10^6^	5.84 × 10^6^	2.71

Mn indicates number average; Mw indicates molecular weight; PD indicates polydispersity index; NS, PDA 0 h, PDA 5 h, PDA 10 h, PDA 15 h, PDA 20 h, and PDA 25 h indicate native starch, pullulanase debranching in different treatment times (h) combined with autoclaving starch.

**Table 3:** Crystallinity patterns and amylose content of native and debranched combined with autoclaved starches.

Samples	Relative Crystallinity (%)	Crystallinity Type	Diffraction Peaks (^O^)	Amylose Content (%)
NS	26.46 ± 1.14 ^d^	C	15.12, 17.08, 17.88, 22.96	21.53 ± 0.37 ^a^
PDA 0 h	29.00 ± 1.07 ^c^	B	14.26, 15.06, 17.10, 22.18, 24.02	16.55 ± 0.07 ^b^
PDA 5 h	30.59 ± 1.78 ^bc^	B	14.08, 14.94, 17.02, 22.08, 23.94	11.37 ± 0.11 ^c^
PDA 10 h	31.08 ± 1.05 ^bc^	B	14.02, 14.92, 17.02, 23.94	9.33 ± 0.17 ^d^
PDA 15 h	32.34 ± 0.04 ^ab^	B	14.04, 15.02, 17.04, 22.02, 23.92	8.34 ± 0.15 ^e^
PDA 20 h	32.45 ± 0.13 ^bc^	B	14.08, 14.96, 17.04, 22.02, 23.92	8.90 ± 0.12 ^d^
PDA 25 h	35.23 ± 1.03 ^a^	B	14.06, 15.06, 17.04, 22.02, 23.96	8.99 ± 0.12 ^d^

Data are mean ± standard deviations, *n* = 3. Values in the same column with different letters are significantly different (*p* < 0.05); NS, PDA 0 h, PDA 5 h, PDA 10 h, PDA 15 h, PDA 20 h, and PDA 25 h indicate native starch and pullulanase debranching in different treatment times (h) combined with autoclaving starch.

**Table 4:** Thermal properties of native and debranched time combined with autoclaved starches.

Samples	Melting Temperature (°C)				
	To	Tp	Tc	Tc–To	ΔH (J/g)
NS	59.00 ± 0.10 ^f^	75.80 ± 0.20 ^d^	84.73 ± 0.23 ^g^	25.60 ± 0.40 ^f^	11.47 ± 0.06 ^d^
PDA 0 h	60.70 ± 0.10 ^e^	75.13 ± 0.04 ^e^	106.82 ± 0.44 ^e^	46.12 ± 0.25 ^cd^	9.68 ± 0.02 ^f^
PDA 5 h	68.63 ± 0.18 ^d^	78.86 ± 0.01 ^c^	114.32 ± 0.42 ^c^	45.69 ± 0.07 ^d^	12.26 ± 0.06 ^c^
PDA 10 h	73.05 ± 0.21 ^c^	78.74 ± 0.10 ^c^	121.95 ± 0.13 ^b^	48.90 ± 0.21 ^b^	12.45 ± 0.28 ^bc^
PDA 15 h	75.40 ± 0.13 ^b^	95.50 ± 0.05 ^a^	127.20 ± 0.27 ^a^	51.80 ± 0.05 ^a^	13.23 ± 0.09 ^a^
PDA 20 h	78.56 ± 0.06 ^a^	88.60 ± 0.06 ^b^	113.38 ± 0.06 ^d^	34.82 ± 0.09 ^e^	12.87 ± 0.08 ^ab^
PDA 25 h	48.30 ± 0.54 ^g^	72.30 ± 0.28 ^d^	95.33 ± 0.14 ^f^	47.03 ± 0.96 ^c^	10.92 ± 0.26 ^e^

Data are mean ± standard deviations, *n* = 3. Values in the same column with different letters are significantly different (*p* < 0.05); NS, PDA 0 h, PDA 5 h, PDA 10 h, PDA 15 h, PDA 20 h, and PDA 25 h indicate native starch and pullulanase debranching in different treatment times (h) combined with autoclaving starch.

**Table 5:** Swelling and solubility of native and modified purple sweet potato starches.

Samples	65 °C	75 °C	85 °C	95 °C
**Swelling Power (g/g)**				
NS	28.52 ± 3.14 ^a^	29.09 ± 1.75 ^a^	29.85 ± 0.62 ^a^	30.32 ± 2.02 ^a^
PDA 0 h	14.92 ± 0.54 ^b^	15.63 ± 0.10 ^b^	15.91 ± 0.33 ^b^	16.64 ± 1.18 ^b^
PDA 5 h	8.17 ± 1.43 ^c^	8.45 ± 0.59 ^c^	8.95 ± 0.73 ^c^	9.27 ± 0.77 ^c^
PDA 10 h	6.66 ± 0.76 ^c^	7.04 ± 0.82 ^c^	7.23 ± 0.49 ^de^	7.66 ± 0.30 ^c^
PDA 15 h	5.63 ± 0.18 ^c^	6.27 ± 0.70 ^c^	6.46 ± 0.34 ^e^	7.00 ± 0.60 ^c^
PDA 20 h	6.54 ± 0.68 ^c^	6.80 ± 0.38 ^c^	6.94 ± 0.63 ^e^	6.90 ± 0.58 ^c^
PDA 25 h	7.66 ± 0.95 ^c^	8.29 ± 0.15 ^c^	8.85 ± 0.76 ^cd^	9.00 ± 0.84 ^c^
**Soluble Solids (%)**				
NS	0.32 ± 0.02 ^b^	0.33 ± 0.03 ^d^	0.37 ± 0.06 ^d^	0.41 ± 0.00 ^d^
PDA 0 h	0.48 ± 0.05 ^bc^	0.48 ± 0.04 ^c^	0.48 ± 0.01 ^c^	0.47 ± 0.04 ^cd^
PDA 5 h	0.40 ± 0.04 ^cd^	0.46 ± 0.00 ^c^	0.48 ± 0.02 ^c^	0.51 ± 0.02 ^c^
PDA 10 h	0.45 ± 0.05 ^bc^	0.45 ± 0.00 ^c^	0.47 ± 0.00 ^c^	0.51 ± 0.04 ^c^
PDA 15 h	0.51 ± 0.03 ^b^	0.52 ± 0.01 ^c^	0.53 ± 0.01 ^c^	0.54 ± 0.03 ^c^
PDA 20 h	0.62 ± 0.01 ^a^	0.63 ± 0.01 ^b^	0.62 ± 0.00 ^b^	0.63 ± 0.02 ^b^
PDA 25 h	0.66 ± 0.02 ^a^	0.73 ± 0.02 ^a^	0.78 ± 0.04 ^a^	0.85 ± 0.01 ^a^

Data are mean ± standard deviations, *n* = 3. Values in the same column with different letters are significantly different (*p* < 0.05); NS, PDA 0 h, PDA 5 h, PDA 10 h, PDA 15 h, PDA 20 h, and PDA 25 h indicate native starch and pullulanase debranching in different treatment times (h) combined with autoclaving starch.

**Table 6:** In vitro digestion properties of native and debranching combined with autoclaving starches.

Samples	Starch Fractions (%)		
	RDS	SDS	RS
NS	13.42 ± 0.16 ^e^	26.06 ± 0.01 ^a^	60.51 ± 0.00 ^e^
PDA 0 h	30.34 ± 0.05 ^a^	13.58 ± 0.05 ^f^	56.07 ± 0.00 ^g^
PDA 5 h	16.63 ± 0.13 ^c^	21.01 ± 0.15 ^b^	62.36 ± 0.00 ^d^
PDA 10 h	15.46 ± 0.16 ^d^	19.55 ± 0.61 ^c^	64.98 ± 0.00 ^c^
PDA 15 h	13.52 ± 0.48 ^e^	16.34 ± 0.63 ^e^	70.14 ± 0.00 ^a^
PDA 20 h	11.96 ± 0.34 ^f^	20.71 ± 0.13 ^bc^	67.32 ± 0.00 ^b^
PDA 25 h	24.96 ± 0.06 ^b^	17.70 ± 0.36 ^d^	57.33 ± 0.05 ^f^

Data are mean ± standard deviations, *n* = 3. Values in the same column with different letters are significantly different (*p* < 0.05); NS, PDA 0 h, PDA 5 h, PDA 10 h, PDA 15 h, PDA 20 h, and PDA 25 h indicate native starch and pullulanase debranching in different treatment times (h) combined with autoclaving starch.

## Data Availability

Not applicable.
